# Abdominal Compartment Syndrome (ACS) With Sigmoid Volvulus (SV): Lost Hours Are Lost Lives

**DOI:** 10.7759/cureus.33741

**Published:** 2023-01-13

**Authors:** Andrea C Marin, Sharon Hechter, Ankita Prasad, Dina Alnabwani, Charles Lwoodsky, Pramil Cheriyath

**Affiliations:** 1 Internal Medicine, Hackensack Meridien Health Ocean Medical Center, Brick, USA; 2 Internal Medicine, Hackensack Meridian Health Ocean Medical Center, Brick, USA; 3 Internal Medicine, Hackenasck Meridian Health Ocean Medical Center, Brick, USA; 4 Internal Medicine, Hackensack Meridian Health, Ocean Medical Center, Brick, USA

**Keywords:** prognosis, ischemia, sepsis, shoch, peritonitins, perforation, gangrene, intraabdominal pressure, anterior compartment syndrome, sigmoid volvulus

## Abstract

Normal intra-abdominal pressure (IAP) ranges from 0 to 5, and abdominal compartment syndrome (ACS) occurs when a sustained IAP >20 mmHg causes organ dysfunction. ACS mainly occurs in patients who are critically ill. It occurs due to an injury or disease in the abdomen or pelvic area, including trauma, abdominal surgery, acute pancreatitis, pancreatic ileus, volvulus, fecal impaction, and ruptured abdominal aortic aneurysm. If not recognized early, ACS leads to multiorgan dysfunction, shock, and sepsis and has high morbidity and mortality. Our patient was brought to the emergency department (ED) following cardiac arrest and resuscitation and was diagnosed with sigmoid volvulus (SV) and ACS. SV is seen in older men, and its presentation is often insidious and leads to bowel gangrene and ACS. The patient's delay in presenting to the hospital and the severity of his condition leads to a poor outcome despite surgery. A delay in recognizing ACS can lead to a worse outcome.

## Introduction

A sigmoid volvulus (SV) is the torsion of a fluid-filled sigmoid colon (SC) around its mesentery, causing intestinal obstruction. It is uncommon in the United States and accounts for only 10% of intestinal obstructions [1]. Outside of the United States, it is a frequent cause of intestinal obstruction in adults [2] and is supposed to be due to a high-fiber diet. It usually occurs in the elderly, with a mean age of 70, and has a male predominance [1]. Neurological or psychiatric illnesses and constipation are present in most patients [3]. Its onset is often insidious, with increasing abdominal pain, nausea, distention, and constipation over three to four days, and older adults frequently present only later [4]. The abdomen is usually distended on physical examination with some tenderness and an empty left iliac fossa [4]. In the early stages of the disease, there is no fever, tachycardia, low blood pressure, guarding of the abdomen, rigidity, or rebound tenderness. If any of these are present, it means perforation and peritonitis. Abdominal imaging is usually confirmatory; an abdomen X-ray may show a dilated SC and the absence of rectal gas. CT images show a whirl pattern due to a dilated SC around its mesocolon and vessels and a bird's-beak appearance of the colonic segments. Failure to recognize it early can lead to mesenteric ischemia, bowel wall perforation, gangrene, and resulting peritonitis, and increasing intra-abdominal pressure (IAP) leads to abdominal compartment syndrome (ACS) [5].

## Case presentation

**Figure 1:**
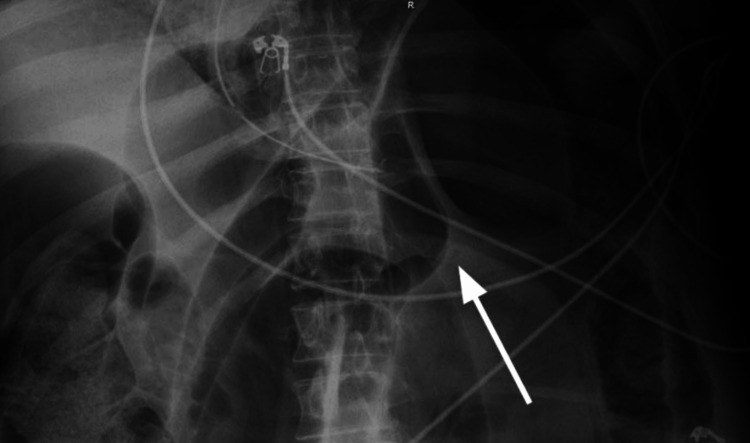
X-ray abdomen showing dilated colon (white arrow).

A 66-year-old man was brought to the emergency department (ED) after being resuscitated by emergency medical personnel for cardiac arrest following a collapse at home. He had worsening abdominal pain and constipation for more than four days, for which he did not seek medical help. He started having abdominal distension and respiratory distress for one day and collapsed at home. He had a history of stroke and hypertension; he was on atorvastatin, aspirin, metoprolol, and losartan for a long time. His family and other personal history were noncontributory, and a review of systems could not be performed due to his unresponsive state. He was intubated at presentation, with good bilateral breath sounds and no signs of trauma; his heart rate was 54 beats per minute, blood pressure was 75/52 mmHg, respiration rate was 20 beats per minute, and oxygen saturation was 89%. His cardiovascular examination showed normal rate, rhythm, and jugular venous pulse; On chest examination, he had normal air entry in both lung fields. He had abdominal distension, and the bowel sounds were absent. He was in severe metabolic acidosis, lactic acidosis, and acute renal failure at presentation. Blood glucose levels were 179 mg/dL, Troponin-0.02 ng/mL (<0.04 ng/mL), arterial blood gases were pH 7.026, pCO2 64.6, paO2 91.8, and bicarbonate was 16.5. A complete blood count revealed leukocytosis 12700 cells/mm*3 (4000-11000/mm*3) with a bandemia-13% (<10%), hemoglobin and platelet count were normal, blood urea nitrogen was 30 mg/dL (6-24 mg/dL), serum creatinine was 2.29 mg/dL (0.6-1.2 mg/dL), and the anion gap was 13. Lactic acid levels were 5.9 (<2.2), and the prothrombin time/international normalized ratio was 14.4 s/1.28; an X-ray abdomen showed massive distension of the large intestines, with intestinal loops compressing the chest cavity, more on the left than the right (Figure 1).

**Figure 2:**
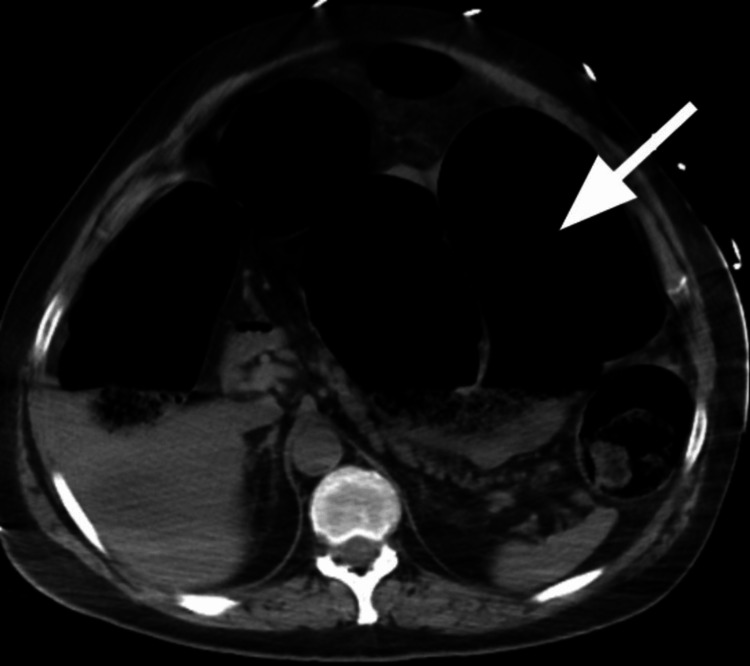
CT abdomen axial view showing massively dilated colon (white arrow).

CT of the abdomen and pelvis revealed distal colonic obstruction with dilated SC secondary to SV and bibasilar atelectasis (Figure 2).

**Figure 3:**
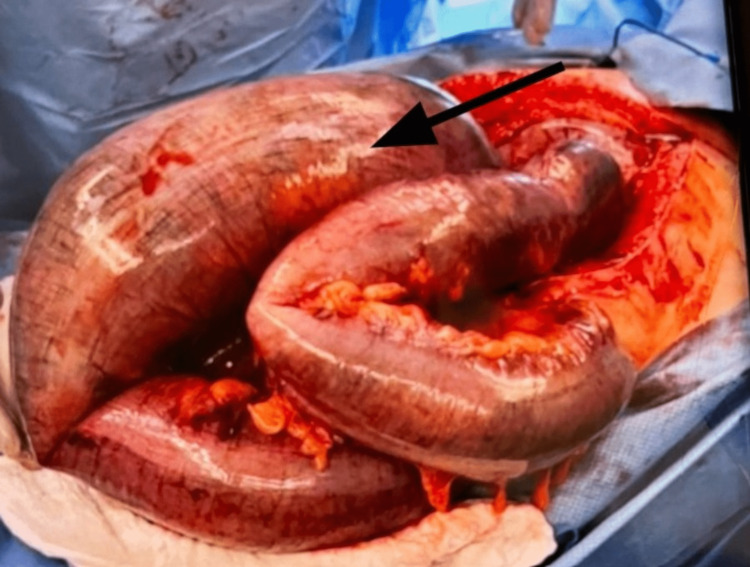
Gangrenous massively dilated descending colon and sigmoid colon (black arrow).

The CT of the head showed a chronic right middle cerebral artery infarct. He received 2000 mL of normal saline before he was started on norepinephrine, later he also required vasopressin and dobutamine infusions. A baseline intravesical measurement of IAP showed a pressure of 44 mmHg. He was diagnosed with SV with peritonitis and ACS leading to multiorgan failure. He was started on piperacillin/tazobactum and taken for an emergency laparotomy and abdominal decompression. During surgery, he was found to have a gangrenous sigmoid and a left colon sigmoidectomy, a left hemicolectomy, Hartmann’s procedure, and a Pico wound, and vacuum-assisted closure (VAC) placement was done (Figure 3).

He had minimal clinical improvement in the post-surgery period and continued to worsen and require ventilatory and pressor support for the next 24 h. He became hemodynamically unstable and eventually passed away. Blood cultures later came positive for *Clostridium hathewayi*.

## Discussion

Due to the SC's torsion along the mesocolon's axis, SV develops and causes a blockage of the large intestine. This obstructs the blood supply to the affected area causing ischemia, gangrene, perforation, and peritonitis. The dilated loops of the colon cause an increased IAP and compression of the vital structures in the abdomen. Increasing pressure on the inferior vena cava reduces venous return and cardiac output, resulting in hypotension. The upward pressure on the diaphragm leads to decreased lung volume, increased peak pressure, and hypercarbia. Compression of both renal veins can result in a reduced glomerular filtration rate, poor urine production, and renal failure. This patient presented with the above complications. In a study on colonic volvulus, the death rate for SV was 9.44% [1]. Patients who develop gangrene have the highest related mortality rate [1]. ACS is a severe condition that affects people who are very sick. Normal IAP ranges from 0 to 5, and ACS occurs when a sustained IAP >20 mmHg causes organ dysfunction. It usually happens after trauma, surgery on the abdomen, or a disease that affects the organs in the abdomen and pelvis. It is regarded as an independent predictor of mortality, making its early detection crucial [6]. The medical care of ACS includes enhancing abdominal wall compliance with decreased muscle contraction, evacuation of luminal contents by decompression by nasogastric tube, evacuation of abdominal fluid by drainage, and correction of positive fluid balance with goal-directed volume resuscitation. It is managed by controlling pain, sedation, paralysis, ventilation, hemodynamic support, and percutaneous surgical decompression. Surgical decompression is the mainstay of treatment for ACS. As most organ dysfunction is a consequence of abdominal compression, organ dysfunction also improves rapidly following surgical abdominal decompression. Using non-surgical therapies early on may prevent IAH from evolving into ACS. The treatment for ACS consists of intraluminal evacuation, intra-abdominal evacuation, enhancement of abdominal wall compliance, fluid management, and enhanced organ perfusion [7]. If ACS is not treated, it can lead to organ failure in multiple systems and death. Both intensive care mortality and 90-day mortality are significantly higher in intra-abdominal hypertension (4.8% and 15.2%, respectively) and ACS (16.7% and 38.9%) than in normal intra-abdominal pressure (1.1% and 7.1%) [8].

Sigmoid volvulus with complicated gangrene can indeed cause these complications, but we did do an intravesical abdominal pressure monitoring to confirm when we suspected ACS. The IAP pre-surgery was 44 mmHg. By definition ACS is an intraabdominal hypertension greater than 20 mm along with new onset organ dysfunction or failure [9]. In our patient in addition to a sustained abdominal pressure >40 mmHg there was evidence of new onset renal failure with blood urea nitrogen of 30 mg/dL (6-24 mg/dL) and serum creatinine was 2.29 mg/dL (0.6-1.2 mg/dL). We started him on piperacillin/tazobactam. So, ACS was the outcome of SV with gangrene. These processes are not exclusive of each other. Two important problems arise in SV: luminal obstruction and vascular occlusion [10]. Both mechanical obstruction and bacterial fermentation cause the distention of the twisted-loop and the proximal colon. Increased intracolonic pressure decreases capillary perfusion [10]. Mucosal ischemic injury causes bacterial translocation and toxemia, resulting in colonic gangrene. Increased intra-abdominal pressure causes ACS [10]. Raveenthiran et al. recently provided more insight into the pathophysiology of acute SV. Increasing intraluminal pressure impairs capillary perfusion following the occurrence of acute SV [11]. Mechanical obstruction due to twisting of mesenteric vessels and thrombosis of mesosigmoid veins contribute to ischemia. Ischemic injury in the mucosa occurs earlier than in other colonic layers and facilitates bacterial translocation and toxemia [6]. A competent ileo-cecal valve converts the proximal colon into a second “closed loop.” Increased intra-abdominal pressure results in the ACS [6]. Prompt and optimal correction of these pathophysiological features is vital to improve the prognosis of acute SV [6, 11].

Our patient presented to the hospital late, i.e., four days after he started with pain abdomen and constipation. In addition, he had already sustained a cardiac arrest at home and was moribund even after resuscitation. Considering the seriousness of his condition, surgical treatment was the only option. Considering his unstable clinical situation, the surgical outcome was also poor. Thus time is of the essence in the management of ACS,

## Conclusions

Sigmoid volvulus mainly develops in elderly patients with neuropsychiatric problems. It Is of insidious onset, which makes the patients present up to three to four days after the start. This delay may lead to gangrene, perforation of the colon, and the development of ACS. The management of ACS involves reducing IAP through medical and surgical methods. Failure to timely manage SV, and ACS is associated with inevitable death due to multi-organ failure.
